# Adverse childhood experience and depression: the role of gut microbiota

**DOI:** 10.3389/fpsyt.2024.1309022

**Published:** 2024-04-02

**Authors:** Yu Bai, Chang Shu, Ying Hou, Gao-Hua Wang

**Affiliations:** ^1^ Department of Psychiatry, Renmin Hospital of Wuhan University, Institute of Neuropsychiatry, Renmin Hospital of Wuhan University, Wuhan, Hubei, China; ^2^ Peking University China-Japan Friendship School of Clinical Medicine, Department of Neurology, Beijing, China

**Keywords:** adverse childhood experiences, gut-brain axis, depressive disorder, gut microbiota, stress

## Abstract

Depression is the most common psychiatric disorder that burdens modern society heavily. Numerous studies have shown that adverse childhood experiences can increase susceptibility to depression, and depression with adverse childhood experiences has specific clinical-biological features. However, the specific neurobiological mechanisms are not yet precise. Recent studies suggest that the gut microbiota can influence brain function and behavior associated with depression through the “microbe-gut-brain axis” and that the composition and function of the gut microbiota are influenced by early stress. These studies offer a possibility that gut microbiota mediates the relationship between adverse childhood experiences and depression. However, few studies directly link adverse childhood experiences, gut microbiota, and depression. This article reviews recent studies on the relationship among adverse childhood experiences, gut microbiota, and depression, intending to provide insights for new research.

## 1 Introduction

Depression, also referred to as major depressive disorder (MDD), is one of the most severe and common psychiatry worldwide (1). It’s characterized by a depressed mood or feeling of sadness, loss of interest or pleasure, low self-esteem and energy, feeling helpless and hopeless, non-suicidal self-injury, and even suicide, disrupting daily activity and psychosocial functions (2). Depression is not only a heavy burden on society and the economy but is also one of the leading causes of disability (3). According to statistics, there are 280 million people affected by depression worldwide (4). However, the specific pathogenesis of depression is still unclear, and nearly half of the depressed patients have unsatisfactory treatment outcomes (5). Therefore, it is essential to explore the pathophysiological mechanisms of depression and discover new biomarkers and therapeutic targets for better treatment of depression.

Adverse Childhood Experiences (ACEs), also known as early life stress (ELS), are a group of stressors that arise from specific causes before age 18, such as abuse: physical, emotional, or sexual abuse; neglect: physical or emotional neglect; chronic family dysfunction; or low socioeconomic status (6). ACEs are significant predictors of depression (7). Meta-analyses have found that childhood neglect and emotional abuse are noteworthy precursors to adult depression (8). Moreover, ACEs were not only associated with the severity of depressive symptoms but also with the chronicity of the depressive course (9). However, the potential mechanisms underlying the effects of ACEs on the development of depression have not been clarified.

A growing number of animal experiments and clinical studies have shown that gut microbiota plays a vital role in the development of depression. Gut microbes communicate bidirectionally with the central nervous system via the vagus nerve, the immune-inflammatory system, bacterial metabolites, neurotransmitters, the HPA axis, and yet-unknown pathways (6). Rodent models of depression suggest that gut microbiota is disrupted after depression (10), consistent with clinical findings in patients with depression (11). It is believed that the interaction between gut microbiota and depression is a bidirectional process. On the one hand, depressed patients exhibit altered gut flora composition (12). On the other hand, the transplantation of “depressed gut flora” from depressed patients can lead to anxiety and depression-like behavior in rodents (13). Recent studies have shown that ACEs can also alter the composition and function of the gut microbiota (14, 15). The gut microbiota has been demonstrated to mediate the effects of early life events on adult behavior (16–18). Tan X, et al. preliminarily addressed that translocation of gut microbiota may be one of the mechanisms by which early life stress influences the development of MDD (19). In Recent years, several studies explored the relationship between ACEs, gut microbiota, and depression directly in the clinical population (20, 21), indicating that gut microbiota had a significant mediating effect between ACEs and depression (21). Moreover, the dysregulation of gut microbiota is associated with suicidal behavior (20). However, these current researches were conducted in relatively small sample sizes, without exploring the specific mechanisms by which gut microbes influence the development of ACEs-induced depression, and lacked consideration of influences such as type of ACE, time of ACE onset, and gender of the subjects. Here, to discuss in further depth the relationship between ACEs and MDD from the perspective of gut microbiota, we sorted through the relevant evidence regarding the relationship between ACEs, gut microbiota, and depression, and summarized in detail the gut microbes that have been reported to be disturbed in various ACE- and MDD-related literature at the phylum, family, and genus levels. Furthermore, we explored the potential mechanisms underlying the involvement of ACE-induced gut microbial disturbances in the subsequent MDD episodes from the perspectives of immuno-inflammatory and neuroendocrine pathways, looking forward to providing new insights for future research.

## 2 The role of ACEs and gut microbiota in depression

### 2.1 ACEs are susceptible factors for developing depression

Clinical studies have shown that ACEs are strongly associated with depression. The correlation between ACEs and depressive episodes is significantly higher than the correlation between recent stressful events and depression (19). Meta-analyses showed that ACEs could dramatically increase the risk of depression in minors and were relevant to the type of ACEs, with sexual abuse, physical abuse, death of family members, domestic violence, and emotional abuse significantly correlated with an elevated risk of depression by age 18 (8). The adverse effects of ACEs related to depression can be sustained into adulthood. Children who experience high levels of adversity in childhood display a higher risk of depression and a more severe degree of depression in adulthood, independent of the duration of adversity occurring (22). Furthermore, individuals who were victims of ACEs also exhibited clinical features such as early onset, chronic course, and suboptimal treatment outcomes (23). In preclinical research, early stress also induces depression in rodents and primates in adulthood. However, the underlying mechanisms by which ACEs influence the development of depression have not been fully elucidated (24, 25).

### 2.2 Gut microbiota disturbance and depression

**Figure 1:**
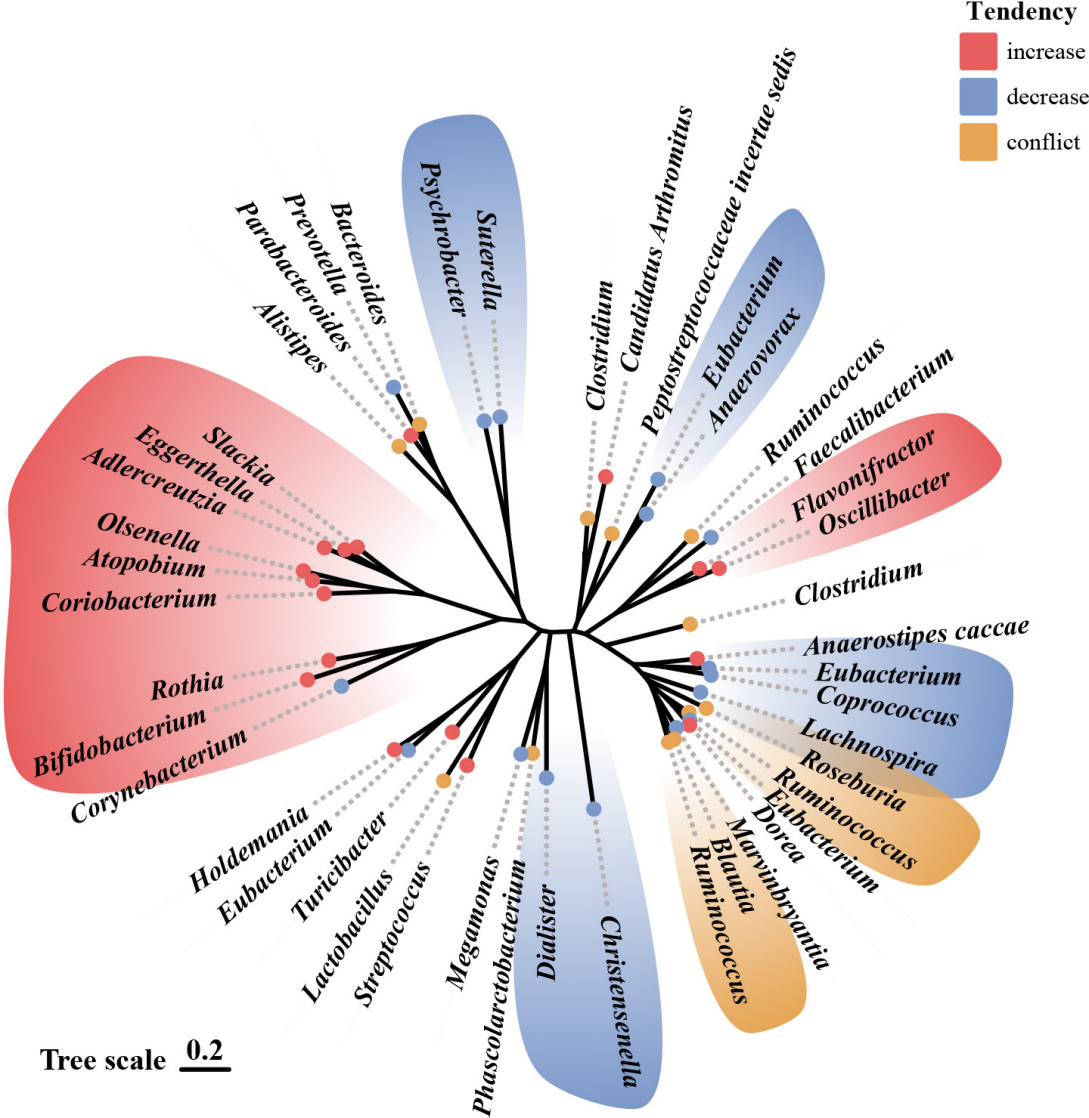
Alterations in gut microbiota observed in studies related to depression.^69^ Illustration of gut microbiota shift associated with depression, based on data presented in **Table 1** and **Table 2**. Genera names are outlined in the figure. Red indicates an increase in abundance, blue indicates a decrease, and yellow indicates that the genus shows different trends in different studies.

Many studies have shown that animals with depressive-like behavior and depressed patients have distinctive gut microbiota characteristics. A recent study using male cynomolgus macaques on the large intestinal mucosal and luminal samples showed that depressed macaques had significantly different in *Firmicutes* and *Bacteroidetes* at the phylum level, as well as *Prevotellaceae* and *Lachnospiraceae* at the family level (26). The gut microbiota composition also differs significantly between depressed patients and healthy controls, but the results are not entirely consistent across studies. At the phylum level, most studies suggest that depressed patients had a significant increase in the relative abundance of the *Bacteroidetes* and *Actinobacteria* relative to controls (27–29). Yet, the results of *Proteobacteria* and *Firmicutes* are highly inconsistent among different studies (27–30). At the family level, nearly 20 taxa were identified in various studies, including *Aminococcaceae*, *Enterobacteriaceae*, *Trichospiraceae*, *Ruminococcaceae*, *Bifidobacteriaceae*, *Erysipelothrichaceae*, *Porphyromonadaceae*, for example (11, 27–34). Among the most consistent findings were a lower abundance of *Prevotellaceae* and *Enterobacteriaceae*, as well as a higher abundance of *Trichosporaceae* in participants with depressive disorders relative to controls (11, 27–31). At the genus level, more than 40 taxa were identified. Most studies showed that short-chain fatty acid-producing bacteria (11, 27, 28, 31, 35–37) such as *Alistipes*, *Oscillospira*, *Faecalibacterium Prausnitzii*, and inflammation-associated bacteria (28, 29, 34, 35, 37–39) such as *Bifidobacterium* and *Roseburia* are significantly associated with depression. Among them, *Faecalibacterium prausnitzii* was shown to be negatively correlated with the severity of depressive symptoms (27, 29) and positively correlated with a higher quality of life (36). **Figure 1** shows the trends in the genera of gut microbiota observed in different studies that explored the development of depression.

Fecal microbiota transplantation in rodents proves that disordered gut microbiota may be an etiological mechanism of depression. Compared to mice transplanted with the “healthy colony,” the depressed mice and mice transplanted with the “depressive colony” from a depressed patient have exhibited depressive-like behavior, increased carbohydrate requirements, and disturbances in amino acid-related metabolites (25). Li et al. (40) also found that the depressed mice and the mice colonized by their gut microbiota exhibited higher levels of anxiety and depressive behavior than the control mice. **Table 1** and **Table 2** provide detailed summaries of the gut microbiota alteration observed in human and animal studies that explore the development of depression, respectively.

**Table 1:** Human studies exploring depression and gut microbiota changes.

Participants	Sample	Methods	Taxonomic differences/Phylum level	Taxonomic differences/Family level	Taxonomic differences/Genus level	Study
37 depressed patients, and 18 HCs	fecal	16S rRNA amplicon sequencing	/	The relative abundance of *Bacteroidales* was increased, while that of *Lachnospiraceae* was decreased in depressed patients compared with that in controls.	*Oscillibacter* and *Alistipes* showed a significant correlation with depression.	Naseribafrouei et al. (2014) (31)
46 depressed patients and 30 HCs (age, 18–40 years)	fecal	PCR and pyrosequencing	In depressed patients, there was a significant increase in the relative abundance of *Bacteroidetes*, *Actinobacteria*, and *Proteobacteria* and a decrease in *Firmicutes*.	The relative abundance of *Enterobacteriaceae* was significantly higher in patients than in controls.	In depressed patients, there was a significant increase in the relative abundance of *Alistipes* and a decrease in that of *Faecalibacterium*. Additionally, the abundance of *Faecalibacterium* was associated with the severity of depressive symptoms.	Jiang et al. (2015) (27)
165 subjects with MDD are compared with 217 BD, and 217 HCs	fecal	16S rRNA amplicon sequencing	MDD groups showed a higher abundance of *Actinobacteria* and a lower abundance of *Bacteroidetes* than HCs.	There was a significant increase in the abundance of *Lactobacteriaceae*, *Streptococcaceae*, *Lachnospiraceae* (*Anaerostipes caccae, Blautia, Dorea*), *Ruminococcaceae*, and a significant decrease in that of *Bacteroidaceae*, *Rikenellaceae*, *Lachnospiraceae* (*Faecalibacterium*), *Acidaminococcaceae*, *Veillonellaceae*, *Sutterellaceae* in MDD groups compared to HCs.	The relative abundance of *Alistipes*, *Coprococcus*, *Clostridium cluster X IV a*, *Roseburia*, *Phascolarctobacterium*, and *Megamonas* was decreased in MDD groups relative to HCs.	Zheng et al. (2016) (25)
10 MDD patients (age, 18–56 years, five women) and 10 HCs (age, 24–65 years, five women)	fecal	Comparative metaproteomics	MDD groups showed an increased proportion of *Actinobacteria* and *Firmicutes*, and a decreased proportion of *Bacteroidetes* and *Proteobacteria* compared to HCs.	In MDD groups, the relative abundance of *Actinomycetaceae*, *Nocardiaceae*, *Streptomycetaceae*, *Bifidobacteriaceae*, *Erysipelotrichaceae*, *Clostridiaceae*, *Lachnospiraceae*, *Ruminococcaceae*, and *Porphyromonadaceae* was increased, whereas that of *Enterobacteriaceae*, *Sutterellaceae*, *Oscillospiraceae*, *Chitinophagaceae*, *Mariniabiliaceae*, *Rikenellaceae*, *Prevotellaceae* was decreased.	The relative abundance of *Faecalibacterium* was significantly lower in depressed patients and negatively correlated with the severity of depression.	Chen et al. (2018) (29)
24 First-episode drug-naïve famale MDD patients and 24 female HCs, 20 First-episode drug-naïve male MDD patients and 20 male HCs	fecal	16S rRNA amplicon sequencing	Compared with HCs, increased *Actinobacteria* and decreased *Bacteroidetes* levels were found in female and male MDD patients, respectively.	Compared to HCs, there is an increase in the relative abundance of *Coriobactericaceae* in female MDD patients; at the same time an increase in that of *Erysipelotrichaceae* and a decrease in that of *Ruminococcaceae* in male MDD patients.	Female MDD patients had higher levels of *Actinomyces*, *Bifidobacterium*, *Acinetobacter*, *Atopobium*, *Eggerthella*, *Gordonibacter*, *Olsenella*, *Eubacterium*, *Anaerostipes*, *Blautia*, *Roseburia*, *Faecalibacterium*, and *Desulfovibrio*, however, lower levels of *Howardella*, *Suterella*, and *Pyramidobacter* than female HCs. Moreover, the relative abundance of *Clostridium cluster XIVa*, *Asaccharobacter*, *Clostridium XIVa*, *Erysipelotrichaceae incertae sedis*, and *Streptococcus* were negatively correlated with the 17-item Hamilton Depression Rating Scale (HDRS) score. For male MDD patients, compared with male HCs, there was an increase in the relative abundance of *Bacteroides*, *Erysipelotrichaceae incertae sedis*, *Veillonella*, and *Atopobium*; in contrast, a decrease in that of *Anaerovorax*, *Gordonibacter*, and *Pyramidobacter*. Moreover, the relative abundance of *Veillonella* was negatively correlated with the 17-HDRS score, and that of *Collinsella* was positively correlated with the 17-HDRS score.	Chen et al. (2018) (29)
36 MDD patients and 37 HCs (age, 20-65 years old)	fecal	16S rRNA amplicon sequencing	MDD patients had higher *Actinobacteria* levels but lower *Bacteroidetes* and *Proteobacteria* levels than HCs.	Compared to HCs, there was an increase in the relative abundance of *Peptostreptococcaceae*, *Porphyromonadaceae*, *Streptococcaceae*, *Bifidobacteriaceae*, *Lachnospiraceae*, whereas a decrease in that of *Alcaligenaceae*, *Prevotellaceae* in MDD patients.	MDD patients showed a significant increase in the relative abundance of *Clostridium cluster X I*, *Holdemania*, *Adlercreutzia*, *Eggerthella*, *Parabacteroides*, *Streptococcus*, *Ruminococcus*, and *Bifidobacterium*, but a decrease in that of *Megamonas*, *Sutterella*, *Prevotella*. Moreover, *Holdemania* was positively correlated with anxiety levels and stress perception levels in depressed patients and not with controls.	Chung et al. (2019) (34)
31 depressed subjects and 30 HCs	fecal	Shotgun metagenomics sequencing	The relative abundance of *Firmicutes* and *Actinobacteria* was significantly increased, but that of *Bacteroidetes* was significantly decreased in depressed subjects compared to HCs.	/	Compared to HCs, the relative abundance of *Bacteroides*, *Clostridium*, *Bifidobacterium*, *Oscillibacter*, and *Streptococcus* in depressed subjects was increased.	Rong et al. (2019) (37)
80 depressed patients (n=40 undergoing antidepressant treatment) and 70 HCs	fecal	16S rRNA amplicon sequencing + Shotgun metagenomic sequencing	/	/	The relative abundance of *Faecalibacterium* and *Coprococcus* was positively associated with QoL (quality of life) scores. *Coprococcus* and *Dialister* remained significantly decreased after eliminating the effects of antidepressant use. Moreover, *Coprococcus* and *Dialister* were both positively associated with QoL scores.	Valles-Colomer et al. (2019) (36)
155 HCs and 156 patients with MDD (age, 18-65 years)	fecal	Metagenomic analysis	/	/	Compared with HCs, MDD subjects were characterized by a higher abundance of *Bacteroides* and a lower abundance of *Blautia* and *Eubacterium*.	Yang et al. (2020) (41)
165 MDD subjects and 217 HCs	fecal	16S rRNA amplicon sequencing	/	In the MDD group, *Bacteroidaceae* and *Bifidobacteriaceae* were higher than HC, whereas *Enterobacteriaceae* was lower than HC.	/	Zheng et al. (2020) (28)
26 depressed subjects (MDD group) and 29 HCs	fecal	Shotgun metagenomic sequencing	Depressed subjects had significantly higher levels of *Actinobacteria* but lower levels of *Bacteroidetes*.	/	The relative abundance of *Slackia*, *Eggerthella*, *Coriobacterium*, *Olsenella*, *Atopobium*, *Rothia*, and *Bifidobacterium* was significantly higher in the depressed subjects.	Lai et al. (2021) (39)
20 depressed subjects (ten females and ten males) and 20 HCs (14 females and 6 males)	fecal	16S rRNA amplicon sequencing based on single nucleotide exact amplicon sequence variants	/	Depressed subjects had higher levels of *Acidaminococcaceae*, *Coriobacteriaceae*, and *Enterobacteriaceae* but lower levels of *Lachnospiraceae*.	In depressed subjects, there was an increase in the relative bundance of *Blautia sp*, *Alistipes sp*, *Parabacteroides spp*, *Phascolarctobacterium sp*, *Oscillibacter spp*, *Rosburia spp*, *Flavonifractor sp*, and *Holdemania sp*, but a decrease in that of *Faecalibacterium spp*, *Ruminococcus spp*, *Lachnospiraceae spp*, and *Bacteroides spp*.	Stevens et al. (2021) (11)
IRONMET cohort (n=116, 44 HCs, 47 mildly depressed subjects, 25 major depressed subjects) IRONMET longitudinal cohort (After 1-year of follow-up individuals were re-evaluated, n=70)	fecal	whole-genome shotgun sequencing	/	/	Patients with higher Patient Health Questionnaire 9 (PHQ-9) scores had higher levels of *Parabacteroides spp.* and *Acidaminococcus spp.* whereas lower levels of *Bifidobacterium pseudolongum* and species from the butyrate-producing *Lachnospiraceae* family. Longitudinal comparisons of baseline bacterial taxa predictive of the PHQ-9 score one year later disclosed lower levels of *Actinobacteria* (*Bifidobacterium spp.* and *Colinsella spp.*) and *Lachnospiraceae* species and higher levels of *Prevotella* and *Enterobacter* species associated with higher PHQ-9 scores one year late.	Mayneris-Perxachs et al. (2022) (38)

HCs healthy controls,MDD major depressive disorder,BD bipolar disorder,PCR Polymerase chain reaction.

**Table 2:** Animal studies exploring depression and gut microbiota changes.

Strains	Model	Sample	Methods	Taxonomic differences/Phylum level	Taxonomic differences/Family level	Taxonomic differences/Genus level	Study
Male Wistar rats	CVS-induced depression rat model	fecal	16S rRNA Gene Sequencing	/	/	The relative abundances of the bacterial genera *Marvinbryantia*, *Corynebacterium*, *Psychrobacter*, *Christensenella*, *Lactobacillus*, *Peptostreptococcaceae incertae sedis*, *Anaerovorax*, *Clostridiales incertae sedis*, and *Coprococcus* were significantly decreased. In contrast, there is an increase relative abundance of *Candidatus Arthromitus* and *Oscillibacter* in model rats compared with normal controls.	Yu et al. (2017) (42)
Male Wistar rats	Depressive rat models of LH	fecal	16S rRNA Gene Sequencing	The relative abundance of *Actinobacteria* was significantly higher in depressed mice than in controls.	The relative abundances of *Lactobacillaceae*, *Turicibacteraceae*, *Peptostreptococcaceae*, and *Bifidobacteriaceae* were significantly higher in the LH group than in the control group, whereas that of *Clostridiales incertae sedis* was significantly lower in the LH group than in the control group.	The relative abundances of *Lactobacillus*, *Turicibacter*, *Peptostreptococcaceae incertae sedis*, and *Bifidobacterium* were significantly higher in the LH group than in the control group, whereas that of *Clostridiales incertae sedis* was significantly lower in the LH group than in the control group.	Takajo et al. (2019) (43)
Male cynomolgus macaques	CUMS induced depression cynomolgus macaques model	Paired mucosal and luminal samples from cecum, ascending, transverse, and descending colons	16S rRNA Gene Sequencing	Significant differences in *Firmicutes* and *Bacteriodetes* were observed between CUMS and control groups.	The abundance of *Lachnospiraceae* was increased in CUMS animals compared to the control group. There was an increase in 11 asv belonging to *Prevotellaceae* and a decrease in 7 asv belonging to *Prevotellaceae*.	The relative abundance of *Lactobacillus* decreased in the CUMS group.	Teng et al. (2022) (26)

CVS chronic variable stress,LH learned helplessness,CUMS Chronic unpredictable mild stress,asv amplicon sequence variant.

Although these studies could not elucidate the causal relationship between gut microbes and depression, they provide a possibility that altered gut microbiota may contribute to depressive episodes. Further research should focus on the impact of different microbial taxa on the development of depression and the specific mechanisms by which they function through the gut-brain axis.

## 3 Effects of ACEs on gut microbiota

### 3.1 Development of gut microbiota

An essential step in identifying and correcting the microbiota of patients is to understand the characteristics of the healthy microbiota and different microbial ecologies that exist in many situations where the disease is not evident.

Studies have shown the developing gut microbiota undergoes three distinct phases: a developmental phase (3-14 months), a transitional phase (15-30 months), and a stable phase (31-46 months). Among them, five phyla (*Actinobacteria*, *Firmicutes*, *Proteobacteria*, *Bacteroidetes*, and *Verrucomicrobia*) and the Shannon diversity index (one of the alpha diversity indices) changed significantly during the developmental period, and *Bifidobacterium* dominated during this phase; *Proteobacteria*, *Bacteroides*, and Shannon diversity index changed significantly during the transitional phase; all phyla and Shannon diversity index were unchanged during the stable phase, what is more, during this period the *Firmicutes* was the dominant group, and there was a high bacterial diversity (44). These studies suggest that most of the structural and functional development of the gut microbiota occurs in the first three years of life. However, recent longitudinal studies in older children suggest full gut microbiota maturation may take longer.

The findings show that the gut microbiota is not yet maturely established by five years of age. Its diversity, core microbiota, and microbial taxa still develop towards adult-type conformations and show inconsistent developmental patterns in different bacterial phyla (45).

A study compared the gut microbiota of healthy children aged 1-4 years with healthy adults aged 21-60. The results showed that *Clostridium cluster XIVa* was equally predominant in young children and adults and is thus considered to be established at an early age. The groups *Actinobacteria*, *Bacilli*, *Clostridium cluster IV*, and *Bacteroidetes* were more prevalent in young children’s guts than adults. At the same time, the abundance of 26 genera shows significant 3.6-fold (higher or lower) differences between young children and adults. Moreover, the microbiota of young children is less diverse than that of adults (46). Furthermore, studies on gut microbiota in adolescents showed that the gut microbiota of adolescents aged 7 to 12 years still differ from those of adults in composition and function and were more complex than those of adults. Regarding microbial composition, although children and adults are dominated by *Bacteroidetes* and *Firmicutes*, children have far more abundance of *Firmicutes* and *Actinobacteria* in their intestines and have a higher Shannon diversity index than adults (47). One reason for this phenomenon may be that adolescents aged 7-12 years are in a critical period of growth and development compared to children aged 0-5 years while facing more complex environmental and dietary changes, which may contribute to the increased diversity of the gut microbial community.

These studies suggest that the composition and function of the gut microbiota span a long period from the establishment to full maturity. Physiologically, the gut microbiota is still not established to a mature configuration at the age of 12 years. Environmental disturbances during this crucial period may alter the gut microbiota’s composition and function, causing multiple problems.

### 3.2 ACEs induce changes in the gut microbiota

A growing body of research indicates that ACEs can disrupt the gut microbiota composition in animals and humans, the effect of which can persist into adulthood. **Table 3** and **Table 4** provide detailed summaries of the gut microbiota alteration relevant to ACEs. At the phylum level, studies found that rats treated with Maternal Separation (MS) had a decrease in the abundance of the *Bacteroidetes* and an increase in the abundance of the *Firmicutes* (53, 58). However, in a “two hits” chronic stress mice model where newborn mice were treated with MS and exposed to 4 weeks of chronic variable stress (CVS) as adults, Kuti et al. showed an increase in the abundance of the *Bacteroidetes* and the *Actinobacteria* (56).

**Table 3:** Human studies exploring ACE and gut microbiota changes.

Participants	Types of ACEs	Sample	Measured methods	Taxonomic differences/Phylum level	Taxonomic differences/Family level	Taxonomic differences/Genus level	Study
82 very low birth weight (VLBW) infants in NICU	early life NICU stress	fecal	16S rRNA amplicon sequencing	/	/	NICU stress exposure had a significant effect on *Proteus* and *Veillonella*. Larger stress scores were associated with smaller probabilities of *Proteus* and *Veillonella* being present. When *Proteus* and *Veillonella* were present, largar stress scores were associated with larger values of relative abundances for both genera.	D'Agata et al. (2019) (46)
Pregnant women ages 18-45 years old who were at 20 to 26 weeks gestation	Adverse childhood experience before the age of 18	fecal	16S rRNA amplicon sequencing	/	/	High ACE participants had higher differential abundance of *Prevotella*, and trend toward lower abundance of *Erysipelotrichaceae* (species previously in *Eubacterium* genus) and *Phascolarctobacterium* than low ACE participants.	Hantsoo et al. (2019) (47)
Children and adolescents who had been exposed to early adverse (EA) caregiving experiences (N=115), and who had been raised with their biological families without any report of adverse caregiving (COMP, N = 229)	EA caregiving experiences	fecal	16S rRNA amplicon sequencing	/	/	The unknown genus in the family *Lachnosporaceae* and the other unknown genus and family from the order *Clostridiales* were higher in children from the COMP than EA groups.	Callaghan et al. (2020) (48)
40 children (5- to 7-year-olds)	socioeconomic risk and adverse home environment exposure	fecal	Metagenomic analyses	CCA model revealed that the socioeconomic risk, caregiver behavior and parent-child dysfunction are significantly associated with taxonomic composition, and the socioeconomic risk and caregiver behavior covariates accounted for 22.3% of the total variance in functional composition.			Flannery et al. (2020) (49)
128 right-handed healthy participants (43 males and 85 females)	childhood traumatic and adverse life events that occurred before the age of 18 years old and covers four domains: general trauma, physical punishment, emotional abuse, and sexual abuse	fecal	16S rRNA amplicon sequencing	There were no significant relationships between a history of ELA exposure and microbial alpha diversity, the variation of microbes within a sample, microbial beta diversity, the variation of microbial communities between samples, or relative taxonomic abundance, at either the phylum or genus levels.			Coley et al. (2021) (14)
17 adolescents (13-21 years) who were internationally adopted, previously institutionalized (PI)/18 adolescents reared in birth families (COMP)	early adverse care	fecal	16S rRNA amplicon sequencing	/	/	The relative abundance of *Prevotella*, *Bacteroides*, *Coprococcus*, *Streptococcus* and *Escherichia* was increased in the PI group compared to COMP group.	Reid et al. (2021) (50)

ACE adverse childhood experience, VLBW very low birth weight, NICU Neonatal Intensive Care Unit, PI previously institutionalized, EA early adverse COMP comparison.

**Table 4:** Animal studies exploring ACE and gut microbiota changes.

Strains	Model	Sample	Methods	Taxonomic differences/Phylum level	Taxonomic differences/Family level	Taxonomic differences/Genus level	Study
Serotonin transporter knockout rats	MS	Fecal	16S rRNA amplicon sequencing	MS showed a shift characterized by a decrease in *Bacteroidetes* and an increase in *Firmicutes*.	There are increases in the abundance of *Spirochaetaceae*, *Ruminococcaceae*, and *Clostridiales* in MS.	MS showed a higher abundance of *Oscillospira*, *Paraprevotella*, *Lachnospira*, *Treponema*, *Desulfovibrio*, *Allobaculum*, and *Clostridium* while a lower abundance of *Phascolarctobacterium*.	EI Aidy et al. (2017) (51)
NMRI mice	MS	Fecal	Real-time PCR method	/	/	The relative abundance of *Bifidobacterium bifidum*, *Lactobacillus*, *Clostridium leptum* and *Clostridium coccoides* (but not *Prevotella* and *Bacteroides fragilis*) in MS increased compared to control mice.	Amini-Khoei et al. (2019) (52)
Male Wistar rats	MS	Fecal	16S rRNA amplicon sequencing	/	MS had a significant increase in the abundance of *Lachnospiraceae*, while a decrease in that of *Bacteroidales S24-7*.	MS had a significant increase in the abundance of *Acetitomaculum*, *Lachnospiraceae*, *Escherichia*, *Clostridiales vadinBB60*, *Desulfovibrio*, *Parabacteroides*, while a decrease in that of *Adlercreutzia*, *Enterorhabdus*, *Intestinibacter*, *Turicibacter* and *Bifidobacterium*.	Rincel et al. (2019) (53)
C3H/HeNRj mice	Multi-hit early life adversity	Fecal	16S rRNA amplicon sequencing	/	/	Male exposed to early adversity displayed a significantly lower relative abundance of unclassified *lachnospiraceaes*, unclassified *Porphyromonadaceae* and a higher relative abundance of *Bacteroides*, *Lactobacillus*, *porphyromonas*, *alloprevotella*, unclassified *Firmicutes* members. In female mice that experienced early adversity, the relative abundance of *Mucispirillum* and *Lactobacillus* was significantly decreased compared with controls.	Rincel et al. (2019) (53)
Male C57BL/6J mice	“Two hits” stress paradigm	Fecal	Real time quantitative PCR	Mice exposed to the “two hits” stress paradigm displayed an increased porportion of *Bacteroidetes* and *Proteobacteria* compared to control mice.	/	The proportion of *Clostridium sp.* increased in mice exposed to the “two hits” stress paradigm compared to control mice.	Kuti et al. (2020) (54)
Sprague-Dawley rats	MS	Fecal	16S rRNA amplicon sequencing	Female MS rats a showed lower abundance of *Deferribacteres*.	In both females and males, the relative abundance of *Lachnospiraceae* and *Pasteurellaceae* was decreased, whereas that of the *Bacteroidaceae* was increased. In male MS rats, the abundance of the *Staphylococcaceae* family was significantly decreased.	In both MS females and males, the relative abundance of the *Bacteroides* genus was increased. In male MS rats, the abundance of the *Dorea*, *Robinsoniella*, and *Staphylococcus*, *Staphylococcus* was significantly reduced, and that of *Streptococcus*, *Gracilibactoer* and *Alkalibaculum* genera was increased. In female MS rats, the abundance of the *Blautia*, *Barnesiella*, *Anaerovorax*, and *Mucispirillum* genera was diminished, and that of the *Sporobacter* genus was elevated.	Park et al. (2020) (56)

MS maternal separation, PCR Polymerase chain reaction.

Early studies have shown that MS can lead to significantly lower levels of *Lactobacilli* in infant rhesus monkeys at the family and genus levels (24). MS paradigm increased the populations of *Bifidobacterium bifidum*, *Lactobacillus*, *Clostridium leptum*, and *Clostridium coccoides* (but not *Prevotella* and *Bacteroides fragilis*) in male mice (54). The study by Kuti et al. (56) similarly confirmed that stress leads to an increased abundance of *Clostridium* spp. in the gut of mice.

**Figure 2:**
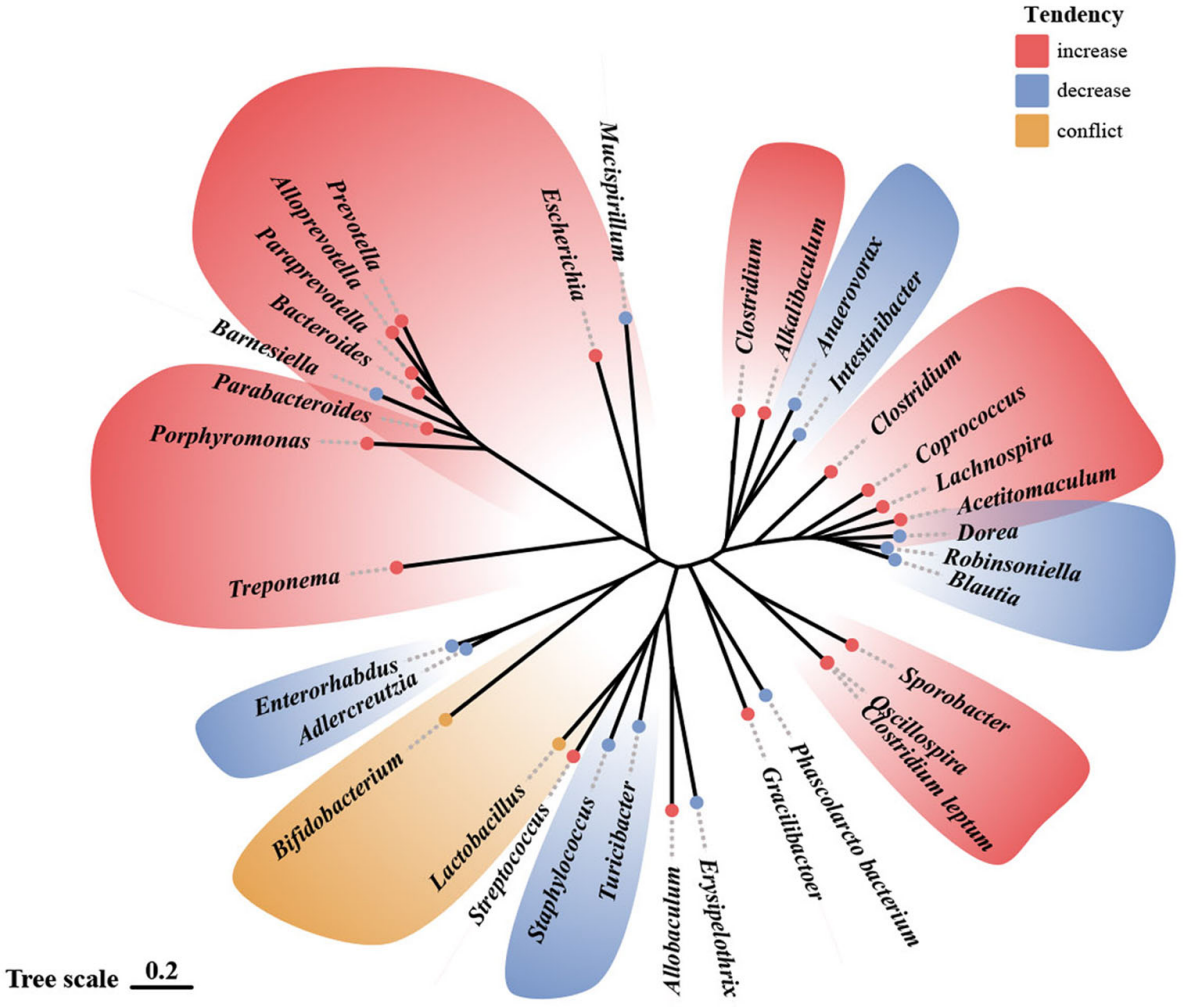
Alterations in gut microbiota observed in human and animal models following ACE exposure.^69^ Illustration of gut microbiota shift following ACE exposure, based on data presented in **Table 3** and **Table 4**. Genera names are outlined in the figure. Red indicates an increase in abundance, blue indicates a decrease, and yellow indicates that the genus shows different trends in different studies.

The difference remained remarkable in healthy individuals versus those who suffered from ACEs. D’Agata et al. (48) conducted a study on preterm infants in NICU. They found that high-intensity stress exposure occurring 1 and 2 weeks before sampling during the first six weeks of life significantly affected *Proteus* and *Veillonella*. Similarly, Flannery et al. (51), showed that socioeconomic risk exposure and behavioral dysregulation in children were associated with the relative abundance of specific gut microbial taxa (e.g., *Bacteroides* and *Bifidobacterium*). A study based on youth aged 5-11 years with exposure to early adverse caregiving experiences (i.e., institutional or foster care followed by international adoption) found that the group with early adverse caregiving experiences had a lower abundance of an unknown genus, which is from the *Lachnospiraceae* of the order *Clostridiales*, than children in the control group (50). This change that ACEs may lead to decreased abundance of *Lachnospiraceae* was likewise confirmed by Rincel et al. (55) and Park et al. (57) In contrast, Reid et al.’s (52) study in 13–21-year-olds with a history of institutional adoption showed that the effects of ACEs (institutional adoption) on the gut microbiota could persist through adolescence and into early adulthood. The relative abundance of *Prevotella*, *Bacteroides* (the family *Bacteroidaceae*), *Streptococcus*, and *Escherichia* was significantly increased within the group with a history of institutional adoption compared to the control group. This variation in the *Bacteroides* was similarly confirmed by Park et al. (57) in a study of rodents. Among mentally healthy pregnant women, high ACE participants had an increased relative abundance of *Prevotella* and a decreased abundance of *Erysipelotrichaceae* and *Phascolarctobacterium* (49). **Figure 2** shows the trends in the genera of gut microbiota observed in different studies associated with ACEs.

The effects of ACEs on rodent gut microbiota may be sex-dependent. Male mice exposed to early life stress had a significantly lower abundance of taxa belonging to *Lachnospiraceae* and *Porphyromonadaceae* and a significantly increased abundance of *Bacteroides*, *Lactobacillus*, *Porphyromonas*, *Prevotella*, and other unclassified Firmicutes. However, only the relative abundance of *Mucispirillum* and *Lactobacillus* was considerably lower in female mice (55). The result above may indicate that the sensitivity of the gut microbiota to early-life stress is higher in male mice than in female mice. Park et al. (57) found an increase in the relative abundance of the *Sporobacter* and a decrease in that of the *Mucispirillum* in female MS rats, whereas the relative abundance of the *Streptococcus* was enhanced and that of the *Staphylococcus* was reduced in male MS rats. The relative abundance of the *Bacteroides* was increased, while that of the *Lachnospiraceae* was decreased in the feces of MS rats of both sexes. These discoveries suggest that MS induces alterations in gut microbes in a sex-dependent manner.

The effect of ACEs on gut microbiota is also related to genotype. Compared to other groups, 5-HTT-/- rats exposed to MS with 5-hydroxytryptamine gene deletion had an increased abundance of microorganisms associated with inflammation, including *Desulfovibrio* and *Clostridium* spp (53).

These studies indicate that ACEs can induce structural and functional alterations in the gut microbiota, which may be a potential pathophysiological pathway for the adverse outcomes induced by ACEs. Future studies should also focus on the effects of different types and degrees of ACEs on specific gut microbial taxa to identify crucial microbial targets of ACEs that induce adverse outcomes.

## 4 Discussion

**Figure 3:**
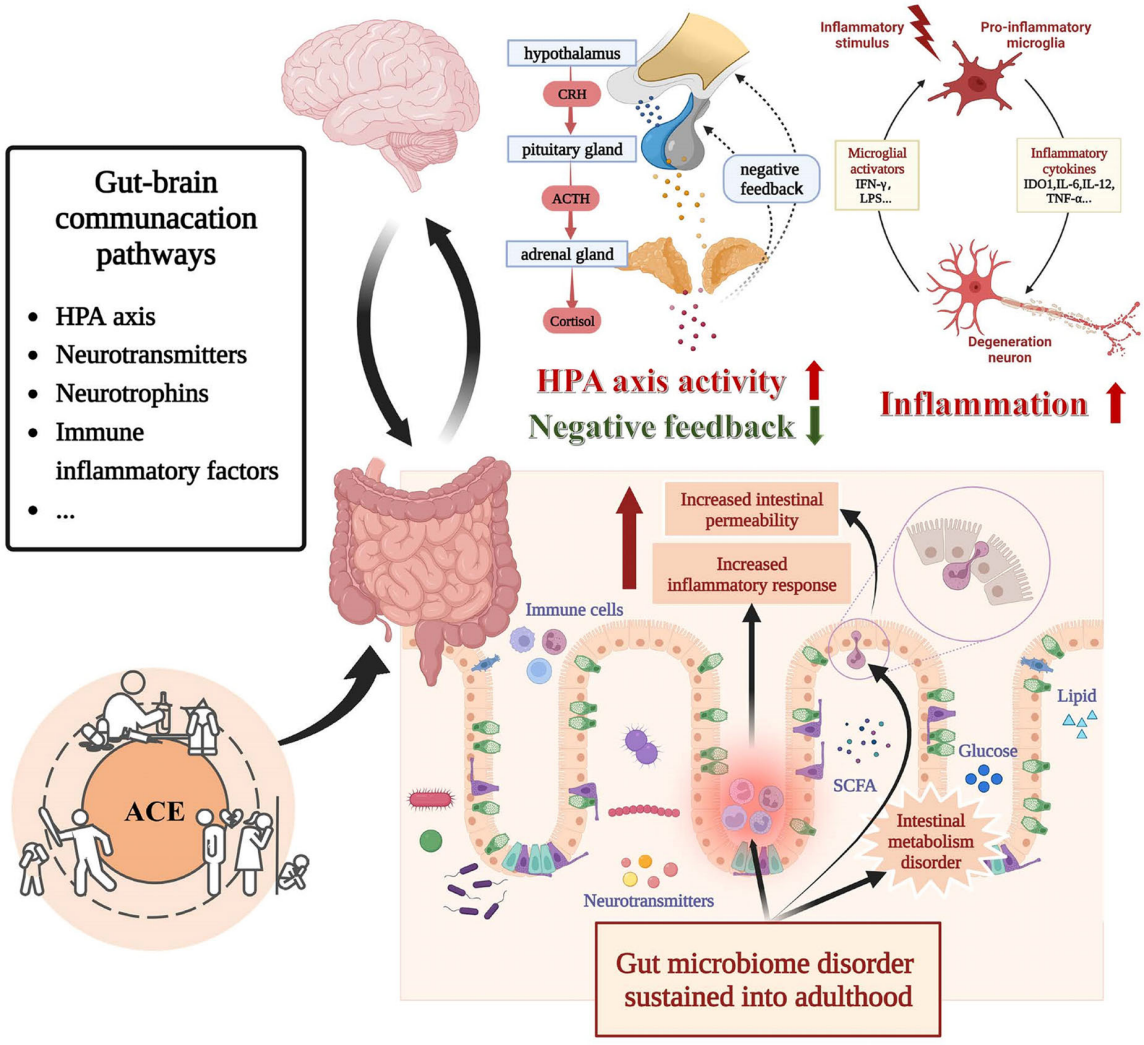
Bidirectional communication between gut microbiota and brain under the influence of ACE. Alterations in the gut microbiota influenced by ACE may lead to alternations in intestinal metabolic, intestinal barrier function, vagus nerve function, HPA function, and immune-inflammatory state. Each of these mechanisms is implicated in the pathophysiology of depressive disorder. Abbreviation: ACE, adverse childhood experience, CRH, corticotropin-releasing hormone, ACTH Adrenocorticotropic Hormone.

This review summarized the evidence provided so far regarding the relationship between ACEs and gut microbiota in depressive disorders, thus facilitating the investigation of the peculiar role of the gut-brain axis in depression associated with ACEs. In the following sections, we will attempt to synthesize the functional relevance of reported microbial taxonomic differences related to ACEs and depression to provide clues for elucidating the mechanisms by which ACE affects depressive disorders through the gut-brain axis. As shown in **Figure 3**, ACEs may influence the bidirectional gut-brain communication primarily through neuroendocrine and immunoinflammatory pathways, thus interfering with the development of depression. We will also reflect on the limitations of the existing literature and then elaborate on important considerations for future research.

### 4.1 Neuroendocrine pathway

Gut microbiota may regulate the development of ACEs-induced MDD through the Neuroendocrine pathway. This process may involve the HPA axis, neurotransmitters and Neurotrophins secreted by the central nervous system, and some metabolites involved in neuroendocrine processes.

#### 4.1.1 The HPA axis

Hyperactivity of the HPA axis is one of the biological markers of depression, and it has been widely suggested that ACEs have long-term effects on the HPA axis. Cortisol arousal responses were increased in participants with ACEs regardless of depression, with depressed patients who experienced ACEs having the highest cortisol concentrations and reduced glucocorticoid negative feedback inhibition after dexamethasone inhibition experiments (59). In the elderly, there were also significant negative associations between ACEs and morning cortisol levels, irrespective of depression (60). This consequent hyperactivity of the HPA axis and negative feedback dysfunction mediated by ACEs may increase susceptibility to depression and poor response to depression treatment in later life.

There is an interaction between HPA axis alterations and disturbances in the gut microbiota associated with ACEs. Individuals experiencing high ACEs showed disturbances in the gut microbiota and blunted glucocorticoid response to stress; moreover, the glucocorticoid response to stress was also positively correlated with the abundance of *Rikenellaceae* and *Dialister* while negatively correlated with that of the *Bacteroides* genus (49). Amini-Khoei et al. (54) showed that adrenalectomy might modulate the harmful effects of MS-induced microbiota disorders and altered inflammatory status, suggesting that the over-activation of the HPA axis may cause MS-induced gut microbiota disorders. The gut microbiota disturbance associated with ACEs is closely related to the dysfunction of the HPA axis, but its specific causal relationship and biological mechanisms need further study to elucidate.

Dysfunction of the HPA axis associated with gut microbiota may play a role in attacks of depression. Multiple studies have shown that plasma adrenocorticotropic hormone and cortisol levels are elevated in germ-free mice after exposure to stress than in specific pathogen-free mice (61–63). At the behavioral level, germ-free mice exhibit lower anxiety and depression (62, 64, 65), whereas “depression microbiota” recipient mice showed anxiety- and depressive-like behaviors (65). Interestingly, expression of GR pathway gene stat5a was downregulated in “depression microbiota” recipient mice, while it was upregulated in germ-free mice (65). These studies indicated that the gut microbiota might lead to behavioral abnormalities in mice through HPA axis responsiveness and the downstream pathway of the glucocorticoid receptor.

#### 4.1.2 Neurotransmitters

Dysfunction in the dopamine (DA) system has been closely linked to anhedonia (66, 67), which is one of the hallmark symptoms of MDD (68). Through activating the HPA axis or inducing epigenetic alterations in neurotransmitter-related genes, ACEs may contribute to the decrease of dopamine in the brain (19, 69), and most relevant studies suggest a downregulation of the DA system in MDD (67). Gut microbiota may modulate the relationship between ACEs, DA, and depression. Compared to the control group, the group of depleted gut microbiota rats processed with antibiotics had higher levodopa (LDOPA) and homovanillic acid (HVA) in the prefrontal cortex, lower HVA in the hippocampus, and lower HVA/DA in the amygdala and striatum. Specific microbiota strains play a beneficial role in the DA system of ELS-induced depression models. It has been shown that *Lactobacillus paracasei PS23* could reduce depression-like behaviors induced by ELS by deregulating the level of 3,4-Dihydroxyphenylacetic acid (DOPAC) and HVA in the hippocampus of male mice (70). Similarly, *Bifidobacterium CECT 7765* has also shown the ability to decrease the level of DA in the hypothalamus and the improvement of the behavior abnormality of the ELS-induced male mice model (71).

Besides DA, the disturbance in the serotonin (5-HT) system has also been considered one of the pathogenic mechanisms of depression (72–74). However, a recent systematic review reported that there is no consistent evidence providing an association between serotonin and depression (75). This finding makes it clear that the current studies cannot prove that lowered 5-HT activity or concentrations can cause depression. Still, it remains evident that 5-HT is involved in multiple functions, including mood, sleep, appetite, and defensive mechanisms (73), and selective serotonin reuptake inhibitors (SSRIs) are still the first-line medication option for depression in clinical practice today (76). It is believed that Gene-environment (GxE) interactions are fundamental elements to the perturbations that occur in the serotonin system (77). Several studies found that polymorphisms of the serotonin-transporter-linked promoter region (5-HTTLPR) could positively moderate the relationship between ACEs and depression (78–85). Likewise, Brezo et al. found that, in the SLC6A4 (Solute carrier family 6, member 4) gene, homozygosity of the A allele of the polymorphism (rs3794808) increased the risk of depression among individuals with childhood physical abuse experiences compared to those who were heterogeneous or homozygous for the G allele (86). gut microbiota can interact with the 5-HT in the brain. In the model of antibiotic-induced depletion of the gut microbiota, male Sprague-Dawley rats exhibited depression-like behavior, higher 5-Hydroxy indoleacetic acid (5-HIAA)/5-HA in the hippocampus, as well as lower 5-HIAA/5-HA in the hypothalamus (87). Another study demonstrated the opposite causality of the association between the serotonin system in the brain and the gut microbiota. EI Aidy et al. (53) found the altered expression of the serotonin transporter (5-HTT) induced dysbiosis in rat gut microbiota, which include the microbial genera *Prevotella*, *Lachnospira, Ruminococcus*, and *Blautia*. Moreover, these 5-HTT genotype-related disruptions increase under the influence of ELS (53).

#### 4.1.3 Neurotrophins

Brain-derived neurotrophic factor (BDNF), a neurotrophic factor expressed in the brain, is involved in various functions including neuronal growth, fear memory extinction, and stress response (88). What’s more, it is a common downstream intermediary for stress factors that potentiate anxiety- and depressive-like behavior (88). In a rodent study, adult rats with MS exhibited disturbance in gut microbiota, a decrease in the level of BDNF, and depressive-like behavior (89). Likewise, Male Sig-1R knockout (KO) mice showed a highly similar depressive-like phenotype (90).Li et al. further provided a possible causal relationship between the dysbiosis in gut microbes and the changes in BDNF by processing Fecal microbiota transplantation (FMT) experiments. Compared to the WT-FWT group, the WT-FKO group had a significantly lower level of BDNF and also the display of depressive-like behavior (90), which indicates that specific microbial taxa changes may mediate the decline in BDNF and thereby promote residual depression-like behaviors. However, the results of clinical studies investigating the relationship between ACEs and BDNF are quite heterogeneous. A recent meta-analysis indicates that there is no significant difference in BDNF protein levels between ACE-exposed and unexposed groups (91). This heterogeneity between studies may be related to the age of the sampling, analyte type, and categories of ACE exposure, and future researchers should pay attention to the impact of these factors on the study results.

#### 4.1.4 Other metabolites

Changes in gut microbiota can also contribute to the disruption of metabolites involved in neuroendocrine processes, which may further exacerbate the development of depression. Studies have identified that the levels of γ-aminobutyric acid (GABA) and tryptophan (Trp) metabolism were disrupted in their intestines (39, 41). Furthermore, there is a negative correlation between the abundance of Trp metabolism-related microbial genes and the HAMD scores in the MDD group (39). Concerning glucose metabolism, depressed patients burn more carbohydrates than healthy controls and have multiple active microbial functional pathways related to carbohydrate metabolism (34). A recent rodent study has shown that excessive sugar intake can disrupt brain function, triggering and exacerbating psychiatric symptoms (92). Metabolites of gut microbes, mainly short-chain fatty acids, can also contribute to the regulation of depression. It has been shown that acetate alters the expression of the neurotransmitter 5-hydroxy trptamine3 (5-HT3) receptor (93), which is closely associated with depression. Propionic acid has been revealed to positively affect the central nervous system by increasing the number of enteric-derived regulatory T cells and, thus, transmyelin regeneration (94). Further understanding of the neuroendocrine function of the gut microbiota in ACE-related depressive disorders may point the way to targeted and individualized interventions.

### 4.2 Immune-inflammatory pathway

ACEs can also cause changes in the immune-inflammatory state of the body, and inflammatory activation may particularly emerge in depressed individuals exposed to early stressors. These hypotheses stem from the higher levels of immune-inflammatory factors and upregulated inflammation-related genes observed in depressed patients with ACEs, which are not present in depressed patients without ACEs (95, 96).

The potential for microbiota-mediated inflammation associated with ACEs in depression is indicated by the disturbance in inflammation-associated microbial members. The study by EI Aidy et al. (53) suggests that changes in gut microbes caused by early life stress may be a risk factor for the development of certain inflammatory-related diseases, and restoring homeostasis of the flora with neuroimmunomodulatory functions can prevent the occurrence of excessive stress-induced inflammatory responses thereby preventing the development of certain diseases. In the preclinical study, recipient mice transplanted with “depression microbiota” from depressed mice showed increased levels of interferon γ (IFN-γ), tumor necrosis factor-α (TNF-α), and indoleamine 2,3-dioxygenase 1 (IDO1) in the hippocampus, as well as higher levels of depressive behavior than control mice (40). A systematic review found that depressed patients with ACEs had significantly higher levels of Interleukin-6 (IL-6) and TNF-α than controls or depressed patients without ACEs (96). Interestingly, the area under the curve (AUC) of IL-6 was found to positively correlate with the abundance of *Bacteroides* and negatively correlate with *Clostridiales*, *Lachnospiraceae*, *Dialister*, and *Enterobacteriaceae*, and the TNF-α AUC was positively correlated with the abundance of *Bacteroides*, *Prevotella*, and *Megasphaera* and negatively correlated with *Ruminococcaceae* (49). Most of these microbial taxa mentioned above, associated with IL-6 and TNF-α, are members of *Bacteroidetes* and *Firmicutes*, which have been widely recognized as the most affected bacterial phyla in depression (30, 97). *Lachnospiraceae*, known for its ability of short-chain fatty acids (SCFA) synthesis, was found to be negatively correlated with depression severity (98). Similarly, *Dialister* was depleted in depression, even after correcting for the confounding effects of antidepressants (36). *Prevotella* and *Megasphaera* are emerging probiotics that apparently improve inflammatory responses (99, 100). A recent study found that *Prevotella histicola* may be therapeutically beneficial for depressive disorder by repairing intestinal leakage and inhibiting central inflammation (100).What’s more, disturbance of microbial metabolites, such as SCFA, could induce abnormalities in the blood-brain-barrier permeability and intestinal permeability (47). And increased blood-brain barrier, and intestinal permeability may cause a hyperinflammatory state in the body (27). These results indicate that immune-inflammatory alterations may be a critical pathway for adverse outcomes induced by disturbances in the gut microbiota associated with ACEs. Future research should focus on changes in the relative abundance of inflammation-associated gut microbes in disease states, the consequent changes in microbiota function, and the specific biological mechanisms involved.

Some studies have confirmed that the alterations in gut microbiota caused by ACEs are sex-specific. Male mice exposed to early life stress had a significantly lower abundance of taxa belonging to *Lachnospiraceae* and *Porphyromonadaceae* and a significantly increased abundance of *Bacteroides*, *Lactobacillus*, *Porphyromonas*, and *Prevotella*. However, only the relative abundance of *Mucispirillum* and *Lactobacillus* was significantly lower in female mice (55). The result above may indicate that the sensitivity of the gut microbiota to early-life stress is higher in male mice than in female mice. Park et al. (57) found an increase in the relative abundance of *Sporobacter* and a decrease in that of the *Mucispirillum* in female MS rats, whereas the relative abundance of the *Streptococcus* was enhanced and that of the *Staphylococcus* was reduced in male MS rats. The relative abundance of the *Bacteroides* genus was increased, while that of the *Lachnospiraceae* was in the feces of MS rats of both sexes. Consistently, Park et al. (57) found that ACEs induced more severe systemic inflammation and anxiety-like behaviors in males than females. Depression also exhibits significant gender differences, with women significantly more affected in prevalence, recurrence, symptoms, and co-morbidity patterns (101). Moreover, these gender differences in depression are probably mirrored in differences in response to antidepressant treatments (101, 102). However, there is an inconsistency between the male susceptibility to ACE-associated microbial disturbance and the female susceptibility commonly seen in depression. Thus, it is also essential to investigate whether the gender specificity of ACEs on gut microbes is related to the gender specificity observed in depression to identify pathophysiological mechanisms and find effective treatments.

### 4.3 Summary and prospects

This review reviewed and summarized recent studies on the relationship among ACEs, gut microbiota, and depression. The available literature suggests that ACEs can increase susceptibility to depression across the lifespan and that there is an association between gut microbiota, ACEs, and depression. On the one hand, the gut microbiota may play an essential role in developing and treating depression by acting on the neuroendocrine system, immune-inflammatory pathways, and organismal metabolism. On the other hand, ACEs may affect the composition and function of the gut microbiota. This effect can persist into adulthood with various organismal inflammatory, immune, and neuroendocrine alterations. Based on this body of evidence, it can be hypothesized that the gut microbiota may be a vital mediator of the development of depression induced by ACEs.

Currently, there are many limitations to the research on gut microbiota. Many studies use animal models that do not accurately reflect human characteristics and behaviors, and relatively few studies on humans are usually in small base populations. At the same time, many confounding factors that may affect the gut microbiota are challenging to control, such as abnormal diet, antibiotics, and antidepressants. In addition, the potential mechanisms by which ACEs modulate the composition of the gut microbiota and by which a disordered microbiota affects the brain remain to be clarified. In studies on gut microbiota and depression, current research is based on the correlation of specific gut microbiota and their metabolites with depression. However, these correlations do not prove a causal relationship between microbiota disorders and the occurrence of depression. The insufficient number of studies directly addresses the relationship between ACEs, gut microbiota, and depression.

Therefore, the role of gut microbes in the association between ACEs and depression deserves further investigation. Meanwhile, studies should further focus on the relationship between ACEs and gut microbes across gender and depressive symptoms to reveal the pathophysiological mechanisms of depression and to find new biomarkers for identifying and diagnosing depression as well as new therapeutic targets for reducing the adverse effects of early stress and depression treatment.

## Author contributions

YB: Conceptualization, Data curation, Writing – original draft. CS: Conceptualization, Supervision, Writing – review & editing. YH: Software, Visualization, Writing – original draft. GW: Conceptualization, Supervision, Writing – review & editing.

## References

[1] Abdoli N Salari N Darvishi N Jafarpour S Solaymani M Mohammadi M. The global prevalence of major depressive disorder (MDD) among the elderly: A systematic review and meta-analysis. Neurosci Biobehav Rev. (2022) 132:1067–73. doi: 10.1016/j.neubiorev.2021.10.041 34742925

[2] Li Z Ruan M Chen J Fang Y. Major depressive disorder: advances in neuroscience research and translational applications. Neurosci Bull. (2021) 37:863–80. doi: 10.1007/s12264-021-00638-3 PMC8192601 33582959

[3] COVID-19 Mental Disorders Collaborators. Global prevalence and burden of depressive and anxiety disorders in 204 countries and territories in 2020 due to the COVID-19 pandemic. Lancet. (2021) 398:1700–12. doi: 10.1016/S0140-6736(21)02143-7 PMC8500697 34634250

[4] Depressive disorder (depression) (2023). Available online at: https://www.who.int/news-room/fact-sheets/detail/depression.

[5] Koopman M El Aidy S MIDtrauma consortium. Depressed gut? The microbiota-diet-inflammation trialogue in depression. . Curr Opin Psychiatry. (2017) 30:369–77. doi: 10.1097/YCO.0000000000000350 28654462

[6] Hantsoo L Zemel BS. Stress gets into the belly: Early life stress and the gut microbiome. Behav Brain Res. (2021) 414:113474. doi: 10.1016/j.bbr.2021.113474 34280457 PMC8380711

[7] Childhood Trauma Meta-Analysis Study Group. Treatment efficacy and effectiveness in adults with major depressive disorder and childhood trauma history: a systematic review and meta-analysis. Lancet Psychiatry. (2022) 9:860–73. doi: 10.1016/S2215-0366(22)00227-9 36156242

[8] LeMoult J Humphreys KL Tracy A Hoffmeister JA Ip E Gotlib IH. Meta-analysis: exposure to early life stress and risk for depression in childhood and adolescence. J Am Acad Child Adolesc Psychiatry. (2020) 59:842–55. doi: 10.1016/j.jaac.2019.10.011 PMC11826385 31676392

[9] Nikkheslat N McLaughlin AP Hastings C Zajkowska Z Nettis MA Mariani N. Childhood trauma, HPA axis activity and antidepressant response in patients with depression. Brain Behav Immun. (2020) 87:229–37. doi: 10.1016/j.bbi.2019.11.024 PMC7327513 31794798

[10] Zhang K Fujita Y Chang L Qu Y Pu Y Wang S. Abnormal composition of gut microbiota is associated with resilience versus susceptibility to inescapable electric stress. Transl Psychiatry. (2019) 9:231. doi: 10.1038/s41398-019-0571-x 31530799 PMC6748977

[11] Stevens BR Roesch L Thiago P Russell JT Pepine CJ Holbert RC. Depression phenotype identified by using single nucleotide exact amplicon sequence variants of the human gut microbiome. Mol Psychiatry. (2021) 26:4277–87. doi: 10.1038/s41380-020-0652-5 PMC11549940 31988436

[12] Yang Z Li J Gui X Shi X Bao Z Han H. Updated review of research on the gut microbiota and their relation to depression in animals and human beings. Mol Psychiatry. (2020) 25:2759–72. doi: 10.1038/s41380-020-0729-1 32332994

[13] Kelly JR Borre Y O’ Brien C Patterson E El Aidy S Deane J. Transferring the blues: Depression-associated gut microbiota induces neurobehavioural changes in the rat. J Psychiatr Res. (2016) 82:109–18. doi: 10.1016/j.jpsychires.2016.07.019 27491067

[14] Coley EJL Mayer EA Osadchiy V Chen Z Subramanyam V Zhang Y. Early life adversity predicts brain-gut alterations associated with increased stress and mood. Neurobiol Stress. (2021) 15:100348. doi: 10.1016/j.ynstr.2021.100348 34113697 PMC8170500

[15] Jašarević E Howerton CL Howard CD Bale TL. Alterations in the vaginal microbiome by maternal stress are associated with metabolic reprogramming of the offspring gut and brain. Endocrinology. (2015) 156:3265–76. doi: 10.1210/en.2015-1177 PMC4541625 26079804

[16] Allen L Dwivedi Y. MicroRNA mediators of early life stress vulnerability to depression and suicidal behavior. Mol Psychiatry. (2020) 25:308–20. doi: 10.1038/s41380-019-0597-8 PMC6974433 31740756

[17] Zhao Y Han L Teopiz KM McIntyre RS Ma R Cao B. The psychological factors mediating/moderating the association between childhood adversity and depression: A systematic review. Neurosci Biobehav Rev. (2022) 137:104663. doi: 10.1016/j.neubiorev.2022.104663 35429512

[18] Starr LR Stroud CB Shaw ZA Vrshek-Schallhorn S. Stress sensitization to depression following childhood adversity: Moderation by HPA axis and serotonergic multilocus profile scores. Dev Psychopathol. (2021) 33:1264–78. doi: 10.1017/S0954579420000474 32684200

[19] Tan X Zhang L Wang D Guan S Lu P Xu X. Influence of early life stress on depression: from the perspective of neuroendocrine to the participation of gut microbiota. Aging (Albany NY). (2021) 13:25588–601. doi: 10.18632/aging.v13i23 PMC8714134 34890365

[20] Maes M Vasupanrajit A Jirakran K Klomkliew P Chanchaem P Tunvirachaisakul C. Adverse childhood experiences and reoccurrence of illness impact the gut microbiome, which affects suicidal behaviours and the phenome of major depression: towards enterotypic phenotypes. Acta Neuropsychiatr. (2023) 35:328–45. doi: 10.1017/neu.2023.21 37052305

[21] Zhang Y Zhang R Liu P Wang J Gao M Zhang J. Characteristics and mediating effect of gut microbiota with experience of childhood maltreatment in major depressive disorder. Front Neurosci. (2022) 16:926450. doi: 10.3389/fnins.2022.926450 35774560 PMC9238290

[22] Tracy M Salo M Slopen N Udo T Appleton AA. Trajectories of childhood adversity and the risk of depression in young adulthood: Results from the Avon Longitudinal Study of Parents and Children. Depress Anxiety. (2019) 36:596–606. doi: 10.1002/da.22887 30884010 PMC6602824

[23] Nelson J Klumparendt A Doebler P Ehring T. Childhood maltreatment and characteristics of adult depression: meta-analysis. Br J Psychiatry. (2017) 210:96–104. doi: 10.1192/bjp.bp.115.180752 27908895

[24] Mt B Cl C. Maternal separation disrupts the integrity of the intestinal microflora in infant rhesus monkeys. Dev psychobiol. (1999) 35. doi: 10.1002/(ISSN)1098-2302 10461128

[25] Zheng P Zeng B Zhou C Liu M Fang Z Xu X. Gut microbiome remodeling induces depressive-like behaviors through a pathway mediated by the host’s metabolism. Mol Psychiatry. (2016) 21:786–96. doi: 10.1038/mp.2016.44 27067014

[26] Teng T Clarke G Maes M Jiang Y Wang J Li X. Biogeography of the large intestinal mucosal and luminal microbiome in cynomolgus macaques with depressive-like behavior. Mol Psychiatry. (2022) 27:1059–67. doi: 10.1038/s41380-021-01366-w PMC9054659 34719692

[27] Jiang H Ling Z Zhang Y Mao H Ma Z Yin Y. Altered fecal microbiota composition in patients with major depressive disorder. Brain Behav Immun. (2015) 48:186–94. doi: 10.1016/j.bbi.2015.03.016 25882912

[28] Zheng P Yang J Li Y Wu J Liang W Yin B. Gut microbial signatures can discriminate unipolar from bipolar depression. Adv Sci (Weinh). (2020) 7:1902862. doi: 10.1002/advs.201902862 32274300 PMC7140990

[29] Chen Z Li J Gui S Zhou C Chen J Yang C. Comparative metaproteomics analysis shows altered fecal microbiota signatures in patients with major depressive disorder. Neuroreport. (2018) 29:417–25. doi: 10.1097/WNR.0000000000000985 29432299

[30] Simpson CA Diaz-Arteche C Eliby D Schwartz OS Simmons JG Cowan CSM. The gut microbiota in anxiety and depression - A systematic review. Clin Psychol Rev. (2021) 83:101943. doi: 10.1016/j.cpr.2020.101943 33271426

[31] Naseribafrouei A Hestad K Avershina E Sekelja M Linløkken A Wilson R. Correlation between the human fecal microbiota and depression. Neurogastroenterol Motil. (2014) 26:1155–62. doi: 10.1111/nmo.12378 24888394

[32] Zheng P Wu J Zhang H Perry SW Yin B Tan X. The gut microbiome modulates gut-brain axis glycerophospholipid metabolism in a region-specific manner in a nonhuman primate model of depression. Mol Psychiatry. (2021) 26:2380–92. doi: 10.1038/s41380-020-0744-2 PMC8440210 32376998

[33] Chen JJ Zheng P Liu YY Zhong XG Wang HY Guo YJ. Sex differences in gut microbiota in patients with major depressive disorder. Neuropsychiatr Dis Treat. (2018) 14:647–55. doi: 10.2147/NDT PMC5833751 29520144

[34] Chung YCE Chen HC Chou HCL Chen IM Lee MS Chuang LC. Exploration of microbiota targets for major depressive disorder and mood related traits. J Psychiatr Res. (2019) 111:74–82. doi: 10.1016/j.jpsychires.2019.01.016 30685565

[35] Sanada K Nakajima S Kurokawa S Barceló-Soler A Ikuse D Hirata A. Gut microbiota and major depressive disorder: A systematic review and meta-analysis. J Affect Disord. (2020) 266:1–13. doi: 10.1016/j.jad.2020.01.102 32056863

[36] Valles-Colomer M Falony G Darzi Y Tigchelaar EF Wang J Tito RY. The neuroactive potential of the human gut microbiota in quality of life and depression. Nat Microbiol. (2019) 4:623–32. doi: 10.1038/s41564-018-0337-x 30718848

[37] Rong H Xie XH Zhao J Lai WT Wang MB Xu D. Similarly in depression, nuances of gut microbiota: Evidences from a shotgun metagenomics sequencing study on major depressive disorder versus bipolar disorder with current major depressive episode patients. J Psychiatr Res. (2019) 113:90–9. doi: 10.1016/j.jpsychires.2019.03.017 30927646

[38] Mayneris-Perxachs J Castells-Nobau A Arnoriaga-Rodríguez M Martin M de la Vega-Correa L Zapata C. Microbiota alterations in proline metabolism impact depression. Cell Metab. (2022) 34:681–701.e10. doi: 10.1016/j.cmet.2022.04.001 35508109

[39] Lai WT Deng WF Xu SX Zhao J Xu D Liu YH. Shotgun metagenomics reveals both taxonomic and tryptophan pathway differences of gut microbiota in major depressive disorder patients. Psychol Med. (2021) 51:90–101. doi: 10.1017/S0033291719003027 31685046

[40] Li N Wang Q Wang Y Sun A Lin Y Jin Y. Fecal microbiota transplantation from chronic unpredictable mild stress mice donors affects anxiety-like and depression-like behavior in recipient mice *via* the gut microbiota-inflammation-brain axis. Stress. (2019) 22:592–602. doi: 10.1080/10253890.2019.1617267 31124390

[41] Yang J Zheng P Li Y Wu J Tan X Zhou J. Landscapes of bacterial and metabolic signatures and their interaction in major depressive disorders. Sci Adv. (2020) 6:eaba8555. doi: 10.1126/sciadv.aba8555 33268363 PMC7710361

[42] Yu M Jia H Zhou C Yang Y Zhao Y Yang M. Variations in gut microbiota and fecal metabolic phenotype associated with depression by 16S rRNA gene sequencing and LC/MS-based metabolomics. J Pharm Biomed Anal. (2017) 138:231–9. doi: 10.1016/j.jpba.2017.02.008 28219800

[43] Takajo T Tomita K Tsuchihashi H Enomoto S Tanichi M Toda H. Depression promotes the onset of irritable bowel syndrome through unique dysbiosis in rats. Gut Liver. (2019) 13(3):325–32. doi: 10.5009/gnl18296 30602220 PMC6529174

[44] Stewart CJ Ajami NJ O’Brien JL Hutchinson DS Smith DP Wong MC. Temporal development of the gut microbiome in early childhood from the TEDDY study. Nature. (2018) 562:583–8. doi: 10.1038/s41586-018-0617-x PMC6415775 30356187

[45] Cheng J Ringel-Kulka T Heikamp-de Jong I Ringel Y Carroll I de Vos WM. Discordant temporal development of bacterial phyla and the emergence of core in the fecal microbiota of young children. ISME J. (2016) 10:1002–14. doi: 10.1038/ismej.2015.177 PMC4796939 26430856

[46] Ringel-Kulka T Cheng J Ringel Y Salojärvi J Carroll I Palva A. Intestinal microbiota in healthy U.S. young children and adults–a high throughput microarray analysis. PLoS One. (2013) 8:e64315. doi: 10.1371/journal.pone.0064315 23717595 PMC3662718

[47] Hollister EB Riehle K Luna RA Weidler EM Rubio-Gonzales M Mistretta TA. Structure and function of the healthy pre-adolescent pediatric gut microbiome. Microbiome. (2015) 3:36. doi: 10.1186/s40168-015-0101-x 26306392 PMC4550057

[48] D’Agata AL Wu J Welandawe MKV Dutra SVO Kane B Groer MW. Effects of early life NICU stress on the developing gut microbiome. Dev Psychobiol. (2019) 61:650–60. doi: 10.1002/dev.21826 PMC6588487 30697700

[49] Hantsoo L Jašarević E Criniti S McGeehan B Tanes C Sammel MD. Childhood adversity impact on gut microbiota and inflammatory response to stress during pregnancy. Brain Behav Immun. (2019) 75:240–50. doi: 10.1016/j.bbi.2018.11.005 PMC6349044 30399404

[50] Callaghan BL Fields A Gee DG Gabard-Durnam L Caldera C Humphreys KL. Mind and gut: Associations between mood and gastrointestinal distress in children exposed to adversity. Dev Psychopathol. (2020) 32:309–28. doi: 10.1017/S0954579419000087 PMC6765443 30919798

[51] Flannery JE Stagaman K Burns AR Hickey RJ Roos LE Giuliano RJ. Gut feelings begin in childhood: the gut metagenome correlates with early environment, caregiving, and behavior. mBio. (2020) 11:e02780–19. doi: 10.1128/mBio.02780-19 PMC6974564 31964729

[52] Reid BM Horne R Donzella B Szamosi JC Coe CL Foster JA. Microbiota-immune alterations in adolescents following early life adversity: A proof of concept study. Dev Psychobiol. (2021) 63:851–63. doi: 10.1002/dev.22061 33249563

[53] El Aidy S Ramsteijn AS Dini-Andreote F van Eijk R Houwing DJ Salles JF. Serotonin transporter genotype modulates the gut microbiota composition in young rats, an effect augmented by early life stress. Front Cell Neurosci. (2017) 11:222. doi: 10.3389/fncel.2017.00222 28824378 PMC5540888

[54] Amini-Khoei H Haghani-Samani E Beigi M Soltani A Mobini GR Balali-Dehkordi S. On the role of corticosterone in behavioral disorders, microbiota composition alteration and neuroimmune response in adult male mice subjected to maternal separation stress. Int Immunopharmacol. (2019) 66:242–50. doi: 10.1016/j.intimp.2018.11.037 30500621

[55] Rincel M Aubert P Chevalier J Grohard PA Basso L Monchaux de Oliveira C. Multi-hit early life adversity affects gut microbiota, brain and behavior in a sex-dependent manner. Brain Behav Immun. (2019) 80:179–92. doi: 10.1016/j.bbi.2019.03.006 30872090

[56] Kuti D Winkler Z Horváth K Juhász B Paholcsek M Stágel A. Gastrointestinal (non-systemic) antibiotic rifaximin differentially affects chronic stress-induced changes in colon microbiome and gut permeability without effect on behavior. Brain Behav Immun. (2020) 84:218–28. doi: 10.1016/j.bbi.2019.12.004 31821847

[57] Park HJ Kim SA Kang WS Kim JW. Early-life stress modulates gut microbiota and peripheral and central inflammation in a sex-dependent manner. Int J Mol Sci. (2021) 22:1899. doi: 10.3390/ijms22041899 33672958 PMC7918891

[58] Pusceddu MM El Aidy S Crispie F O’Sullivan O Cotter P Stanton C. N-3 polyunsaturated fatty acids (PUFAs) reverse the impact of early-life stress on the gut microbiota. PLoS One. (2015) 10:e0139721. doi: 10.1371/journal.pone.0139721 26426902 PMC4591340

[59] Lu S Gao W Huang M Li L Xu Y. In search of the HPA axis activity in unipolar depression patients with childhood trauma: Combined cortisol awakening response and dexamethasone suppression test. J Psychiatr Res. (2016) 78:24–30. doi: 10.1016/j.jpsychires.2016.03.009 27049575

[60] Wielaard I Schaakxs R Comijs HC Stek ML Rhebergen D. The influence of childhood abuse on cortisol levels and the cortisol awakening response in depressed and nondepressed older adults. World J Biol Psychiatry. (2018) 19:440–9. doi: 10.1080/15622975.2016.1274829 28120636

[61] Huo R Zeng B Zeng L Cheng K Li B Luo Y. Microbiota modulate anxiety-like behavior and endocrine abnormalities in hypothalamic-pituitary-adrenal axis. Front Cell Infect Microbiol. (2017) 7:489. doi: 10.3389/fcimb.2017.00489 29250490 PMC5715198

[62] Crumeyrolle-Arias M Jaglin M Bruneau A Vancassel S Cardona A Daugé V. Absence of the gut microbiota enhances anxiety-like behavior and neuroendocrine response to acute stress in rats. Psychoneuroendocrinology. (2014) 42:207–17. doi: 10.1016/j.psyneuen.2014.01.014 24636517

[63] Vagnerová K Vodička M Hermanová P Ergang P Šrůtková D Klusoňová P. Interactions between gut microbiota and acute restraint stress in peripheral structures of the hypothalamic-pituitary-adrenal axis and the intestine of male mice. Front Immunol. (2019) 10:2655. doi: 10.3389/fimmu.2019.02655 31798585 PMC6878942

[64] Sudo N Chida Y Aiba Y Sonoda J Oyama N Yu XN. Postnatal microbial colonization programs the hypothalamic-pituitary-adrenal system for stress response in mice. J Physiol. (2004) 558:263–75. doi: 10.1113/jphysiol.2004.063388 PMC1664925 15133062

[65] Luo Y Zeng B Zeng L Du X Li B Huo R. Gut microbiota regulates mouse behaviors through glucocorticoid receptor pathway genes in the hippocampus. Transl Psychiatry. (2018) 8:187. doi: 10.1038/s41398-018-0240-5 30194287 PMC6128920

[66] Der-Avakian A Markou A. The neurobiology of anhedonia and other reward-related deficits. Trends Neurosci. (2012) 35:68–77. doi: 10.1016/j.tins.2011.11.005 22177980 PMC3253139

[67] Belujon P Grace AA. Dopamine system dysregulation in major depressive disorders. Int J Neuropsychopharmacol. (2017) 20:1036–46. doi: 10.1093/ijnp/pyx056 PMC5716179 29106542

[68] Rakel RE. Depression. Prim Care. (1999) 26:211–24. doi: 10.1016/S0095-4543(08)70003-4 10318745

[69] Sasagawa T Horii-Hayashi N Okuda A Hashimoto T Azuma C Nishi M. Long-term effects of maternal separation coupled with social isolation on reward seeking and changes in dopamine D1 receptor expression in the nucleus accumbens *via* DNA methylation in mice. Neurosci Lett. (2017) 641:33–9. doi: 10.1016/j.neulet.2017.01.025 28111354

[70] Liao JF Hsu CC Chou GT Hsu JS Liong MT Tsai YC. Lactobacillus paracasei PS23 reduced early-life stress abnormalities in maternal separation mouse model. Benef Microbes. (2019) 10:425–36. doi: 10.3920/BM2018.0077 30882243

[71] Moya-Pérez A Perez-Villalba A Benítez-Páez A Campillo I Sanz Y. Bifidobacterium CECT 7765 modulates early stress-induced immune, neuroendocrine and behavioral alterations in mice. Brain Behav Immun. (2017) 65:43–56. doi: 10.1016/j.bbi.2017.05.011 28512033

[72] Haleem DJ. Targeting serotonin1A receptors for treating chronic pain and depression. Curr Neuropharmacol. (2019) 17:1098–108. doi: 10.2174/1570159X17666190811161807 PMC7057205 31418663

[73] Pourhamzeh M Moravej FG Arabi M Shahriari E Mehrabi S Ward R. The roles of serotonin in neuropsychiatric disorders. Cell Mol Neurobiol. (2022) 42:1671–92. doi: 10.1007/s10571-021-01064-9 PMC11421740 33651238

[74] Jauhar S Cowen PJ Browning M. Fifty years on: Serotonin and depression. J Psychopharmacol. (2023) 37:237–41. doi: 10.1177/02698811231161813 PMC10076339 36938996

[75] Moncrieff J Cooper RE Stockmann T Amendola S Hengartner MP Horowitz MA. The serotonin theory of depression: a systematic umbrella review of the evidence. Mol Psychiatry. (2023) 28:3243–56. doi: 10.1038/s41380-022-01661-0 PMC10618090 35854107

[76] Kupfer DJ Frank E Phillips ML. Major depressive disorder: new clinical, neurobiological, and treatment perspectives. Lancet. (2012) 379:1045–55. doi: 10.1016/S0140-6736(11)60602-8 PMC3397431 22189047

[77] Palma-Gudiel H Fañanás L. An integrative review of methylation at the serotonin transporter gene and its dialogue with environmental risk factors, psychopathology and 5-HTTLPR. Neurosci Biobehav Rev. (2017) 72:190–209. doi: 10.1016/j.neubiorev.2016.11.011 27880876

[78] Aguilera M Arias B Wichers M Barrantes-Vidal N Moya J Villa H. Early adversity and 5-HTT/BDNF genes: new evidence of gene-environment interactions on depressive symptoms in a general population. Psychol Med. (2009) 39:1425–32. doi: 10.1017/S0033291709005248 19215635

[79] Carli V Mandelli L Zaninotto L Roy A Recchia L Stoppia L. A protective genetic variant for adverse environments? The role of childhood traumas and serotonin transporter gene on resilience and depressive severity in a high-risk population. Eur Psychiatry. (2011) 26:471–8. doi: 10.1016/j.eurpsy.2011.04.008 21684723

[80] Carver CS Johnson SL Joormann J Lemoult J Cuccaro ML. Childhood adversity interacts separately with 5-HTTLPR and BDNF to predict lifetime depression diagnosis. J Affect Disord. (2011) 132:89–93. doi: 10.1016/j.jad.2011.02.001 21420735

[81] Fergusson DM Horwood LJ Miller AL Kennedy MA. Life stress, 5-HTTLPR and mental disorder: findings from a 30-year longitudinal study. Br J Psychiatry. (2011) 198:129–35. doi: 10.1192/bjp.bp.110.085993 PMC3031653 21282783

[82] Brown GW Ban M Craig TKJ Harris TO Herbert J Uher R. Serotonin transporter length polymorphism, childhood maltreatment, and chronic depression: a specific gene-environment interaction. Depress Anxiety. (2013) 30:5–13. doi: 10.1002/da.21982 22847957

[83] Kudinova AY Gibb BE McGeary JE Knopik VS. Brain derived neurotrophic factor (BDNF) polymorphism moderates the interactive effect of 5-HTTLPR polymorphism and childhood abuse on diagnoses of major depression in women. Psychiatry Res. (2015) 225:746–7. doi: 10.1016/j.psychres.2014.10.030 PMC4447084 25500322

[84] Simons JS Simons RM O’Brien C Stoltenberg SF Keith JA Hudson JA. PTSD, alcohol dependence, and conduct problems: Distinct pathways *via* lability and disinhibition. Addict Behav. (2017) 64:185–93. doi: 10.1016/j.addbeh.2016.08.044 PMC5143199 27619010

[85] Lipsky RK McDonald CC Souders MC Carpio CC Teitelman AM. Adverse childhood experiences, the serotonergic system, and depressive and anxiety disorders in adulthood: A systematic literature review. Neurosci Biobehav Rev. (2022) 134:104495. doi: 10.1016/j.neubiorev.2021.12.018 34919986

[86] Brezo J Bureau A Mérette C Jomphe V Barker ED Vitaro F. Differences and similarities in the serotonergic diathesis for suicide attempts and mood disorders: a 22-year longitudinal gene-environment study. Mol Psychiatry. (2010) 15:831–43. doi: 10.1038/mp.2009.19 19381154

[87] Hoban AE Moloney RD Golubeva AV McVey Neufeld KA O’Sullivan O Patterson E. Behavioural and neurochemical consequences of chronic gut microbiota depletion during adulthood in the rat. Neuroscience. (2016) 339:463–77. doi: 10.1016/j.neuroscience.2016.10.003 27742460

[88] Notaras M van den Buuse M. Neurobiology of BDNF in fear memory, sensitivity to stress, and stress-related disorders. Mol Psychiatry. (2020) 25:2251–74. doi: 10.1038/s41380-019-0639-2 31900428

[89] Donoso F Egerton S Bastiaanssen TFS Fitzgerald P Gite S Fouhy F. Polyphenols selectively reverse early-life stress-induced behavioural, neurochemical and microbiota changes in the rat. Psychoneuroendocrinology. (2020) 116:104673. doi: 10.1016/j.psyneuen.2020.104673 32334345

[90] Li JH Liu JL Li XW Liu Y Yang JZ Chen LJ. Gut microbiota from sigma-1 receptor knockout mice induces depression-like behaviors and modulates the cAMP/CREB/BDNF signaling pathway. Front Microbiol. (2023) 14:1143648. doi: 10.3389/fmicb.2023.1143648 37089558 PMC10116000

[91] Vyas N Wimberly CE Beaman MM Kaplan SJ Rasmussen LJH Wertz J. Systematic review and meta-analysis of the effect of adverse childhood experiences (ACEs) on brain-derived neurotrophic factor (BDNF) levels. Psychoneuroendocrinology. (2023) 151:106071. doi: 10.1016/j.psyneuen.2023.106071 36857833 PMC10073327

[92] Hirai S Miwa H Tanaka T Toriumi K Kunii Y Shimbo H. High-sucrose diets contribute to brain angiopathy with impaired glucose uptake and psychosis-related higher brain dysfunctions in mice. Sci Adv. (2021) 7:eabl6077. doi: 10.1126/sciadv.abl6077 34757783 PMC8580307

[93] Bhattarai Y Schmidt BA Linden DR Larson ED Grover M Beyder A. Human-derived gut microbiota modulates colonic secretion in mice by regulating 5-HT3 receptor expression via acetate production. Am J Physiol Gastrointest Liver Physiol. (2017) 313:G80–7. doi: 10.1152/ajpgi.00448.2016 PMC5538830 28408644

[94] Hirschberg S Gisevius B Duscha A Haghikia A. Implications of diet and the gut microbiome in neuroinflammatory and neurodegenerative diseases. Int J Mol Sci. (2019) 20:E3109. doi: 10.3390/ijms20123109 PMC6628344 31242699

[95] Schiweck C Claes S Van Oudenhove L Lafit G Vaessen T de Beeck GO. Childhood trauma, suicide risk and inflammatory phenotypes of depression: insights from monocyte gene expression. Transl Psychiatry. (2020) 10:296. doi: 10.1038/s41398-020-00979-z 32839428 PMC7445278

[96] Gill H El-Halabi S Majeed A Gill B Lui LMW Mansur RB. The association between adverse childhood experiences and inflammation in patients with major depressive disorder: A systematic review. J Affect Disord. (2020) 272:1–7. doi: 10.1016/j.jad.2020.03.145 32379599

[97] Zhang Y Fan Q Hou Y Zhang X Yin Z Cai X. Bacteroides species differentially modulate depression-like behavior *via* gut-brain metabolic signaling. Brain Behav Immun. (2022) 102:11–22. doi: 10.1016/j.bbi.2022.02.007 35143877

[98] Li H Xiang Y Zhu Z Wang W Jiang Z Zhao M. Rifaximin-mediated gut microbiota regulation modulates the function of microglia and protects against CUMS-induced depression-like behaviors in adolescent rat. J Neuroinflammation. (2021) 18:254. doi: 10.1186/s12974-021-02303-y 34736493 PMC8567657

[99] Średnicka P Roszko MŁ Popowski D Kowalczyk M Wójcicki M Emanowicz P. Effect of in *vitro* cultivation on human gut microbiota composition using 16S rDNA amplicon sequencing and metabolomics approach. Sci Rep. (2023) 13:3026. doi: 10.1038/s41598-023-29637-2 36810418 PMC9945476

[100] Huang F Liu X Xu S Hu S Wang S Shi D. Prevotella histicola Mitigated Estrogen Deficiency-Induced Depression *via* Gut Microbiota-Dependent Modulation of Inflammation in Ovariectomized Mice. Front Nutr. (2021) 8:805465. doi: 10.3389/fnut.2021.805465 35155523 PMC8826649

[101] Silveira PP Pokhvisneva I Howard DM Meaney MJ. A sex-specific genome-wide association study of depression phenotypes in UK Biobank. Mol Psychiatry. (2023) 28, 1–11. doi: 10.1101/2022.03.30.22273201 36750733 PMC10611579

[102] Carvalho Silva R Pisanu C Maffioletti E Menesello V Bortolomasi M PROMPT consortium. Biological markers of sex-based differences in major depressive disorder and in antidepressant response. Eur Neuropsychopharmacol. (2023) 76:89–107. doi: 10.1016/j.euroneuro.2023.07.012 37595325

